# Effectiveness of individualized inhaler technique training on low adherence (LowAd) in ambulatory patients with COPD and asthma

**DOI:** 10.1038/s41533-021-00262-8

**Published:** 2022-01-10

**Authors:** Juan Miguel Sánchez-Nieto, Roberto Bernabeu-Mora, Irene Fernández-Muñoz, Andrés Carrillo-Alcaraz, Juan Alcántara-Fructuoso, Javier Fernández-Alvarez, Juan Carlos Vera-Olmos, María José Martínez-Ferre, Mercedes Garci-Varela Olea, Maria José Córcoles Valenciano, Diego Salmerón Martínez

**Affiliations:** 1 Division of Pneumology, Morales Meseguer General University Hospital, 30008 Murcia, Spain; 2 Institute for Bio-health Research of Murcia (IMIB-Arrixaca), El Palmar, 30120 Murcia, Spain; 3 Department of Internal Medicine, University of Murcia, El Palmar, 30120 Murcia, Spain; 4 Division of Intensive Care Unit, Morales Meseguer General University Hospital, 30008 Murcia, Spain; 5 Department of Health and Social Sciences, Murcia University, Murcia, Spain; 6 CIBER Epidemiología y Salud Pública (CIBERESP), Madrid, Spain

**Keywords:** Asthma, Chronic obstructive pulmonary disease

## Abstract

To analyze whether there is improvement in adherence to inhaled treatment in patients with COPD and asthma after an educational intervention based on the teach-to-goal method. This is a prospective, non-randomized, single-group study, with intervention and before-after evaluation. The study population included 120 patients (67 females and 53 males) diagnosed with asthma (70.8%) and COPD (29.1%). The level of adherence (low and optimal) and the noncompliance behavior pattern (erratic, deliberate and unwitting) were determined by the Test of the adherence to Inhalers (TAI). This questionnaire allows you to determine the level of adherence and the types of noncompliance. Low Adherence (LowAd) was defined as a score less than 49 points. All patients received individualized educational inhaler technique intervention (IEITI). Before the IEITI, 67.5% of the patients had LowAd. Following IEITI, on week 24, LowAd was 55% (*p* = 0.024). Each patient can present one or more types of noncompliance. The most frequent type was forgetting to use the inhaler (erratic), 65.8%. The other types were deliberate: 43.3%, and unwitting: 57.5%. All of them had decreased on the final visit: 51.7% (*p* = 0.009), 25.8% (*p* = 0.002), 39.2% (*p* = 0.002). There were no significant differences in adherence between asthma and COPD patients at the start of the study. The only predicting factor of LowAd was the female gender. An individualized educational intervention, in ambulatory patients with COPD and asthma, in real-world clinical practice conditions, improves adherence to the inhaled treatment.

## Introduction

Chronic obstructive pulmonary disease (COPD) and asthma are conditions particularly prone to adherence issues due to their chronic nature and to their periods of symptom remission^1^. Incorrect adherence and inhaler technique reduces the treatment benefits and leads to concerns in the healthcare management and health-related consequences^2^. Adherence to oral or inhaler medication ranges between 41 to 57% in COPD^3,4^ and, in asthma, it is 50% in children^5^ and 30% in adults^6^. Adherence is associated with numerous factors such as the disease, the route of administration, access to the treatment and specific characteristics of the patient^7^. Some systematic reviews have evaluated the effectiveness of interventions to improve medication adherence, from self-management training to eHealth tools, with heterogeneous results^8,9^. Similarly, assessing medication adherence has been done using a variety of methods and has rendered heterogeneous results. There is no standard prospective methodology in COPD or asthma^10^. The stated objectives include biochemical or electronic monitoring of medication administration^11^. An example of these is the audio recording devices which simultaneously report on inhaler technique and adherence^12^. Self-reporting questionnaires overestimate adherence. Also, most of these instruments have been designed to monitor oral medication^13,14^. Recently, the Test of adherence to inhalers (TAI)^15^ has been validated for asthma and COPD. It comprises two complementary 12-item questionnaires with domains for patients and for professionals. It gathers information on the degree of adherence and patterns of noncompliance. This test correlated better with adhesion measures made with electronic devices than the Morisky-Green test^15^.

The inappropriate use of an inhaler is one of the most commonly associated barriers with LowAd. Even easy application devices require training^16^. The ability to successfully administer medication through an inhaler has a direct effect, not just on their deposition but also in the perception of benefits by the patient and in their willingness to maintain adherence. The training of the inhaler technique is the main factor that health professionals can modify, although the real benefits are controversial^17^. The most effective training method to teach the inhaler technique is verbal instruction combined with a physical demonstration^18,19^. The objective of the present study is to evaluate adherence to inhaled treatment using TAI^15^, in real clinical practice conditions, with a cohort of ambulatory patients diagnosed with asthma and COPD; before and after an individualized educational inhaler technique intervention (IEITI).

## Methods

### Study design and participants

The prospective, non-randomized, single-group study, with intervention and before-after evaluation, carried out between January 11, 2017 and December 21, 2018. Were included 160 ambulatory patients from a Pulmonology Department of a Public General University Hospital. The patients included were adults >18 years of age, diagnosed of bronchial asthma or COPD, who were being treated with one of the following devices: Pressurized metered-dose inhaler (pMDI)/Soft mist inhaler (SMI), Dry powder inhaler multidose (DPIm), Dry powder inhaler single dose (DPIs), and Pressurized metered-dose inhaler (pMDI) with spacer holding chamber (pMDI + spacer). The diagnosis of asthma was based on GINA criteria^20^. The diagnosis of COPD was done using GOLD criteria of airflow limitation (FEV1/FVC post-bronchodilator <0,70)^21^. In all cases, more than 6 months have passed since the initial diagnosis of COPD or asthma. Patients over 70 years old and/or with psychiatric history were evaluated for cognitive function using the Pentagon Drawing Test^22^. The patients who did not pass this test were excluded from the study and the treatment with nebulizers was recommended. Other criteria for exclusion were refusal to participate and the presence of a language barrier. The study protocol was approved by the institutional review board of the hospital, called the “Ethical Committee of Clinical Research of the General University Hospital” on 09/28/2016 (approval number: EST-30/16). All study participants provided written informed consent.

**Fig. 1:**
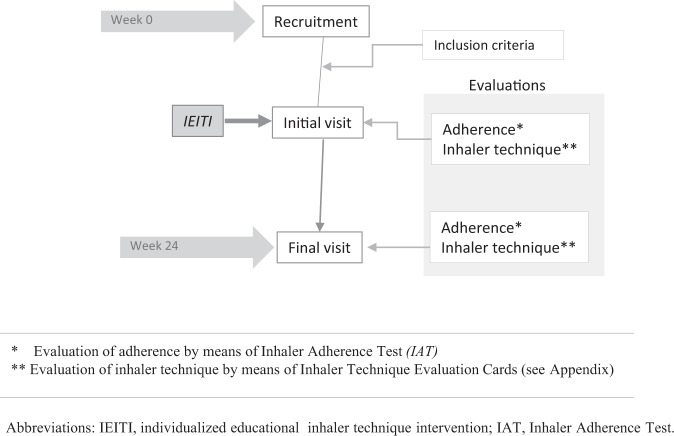
Study sequence.

**Fig. 2:**
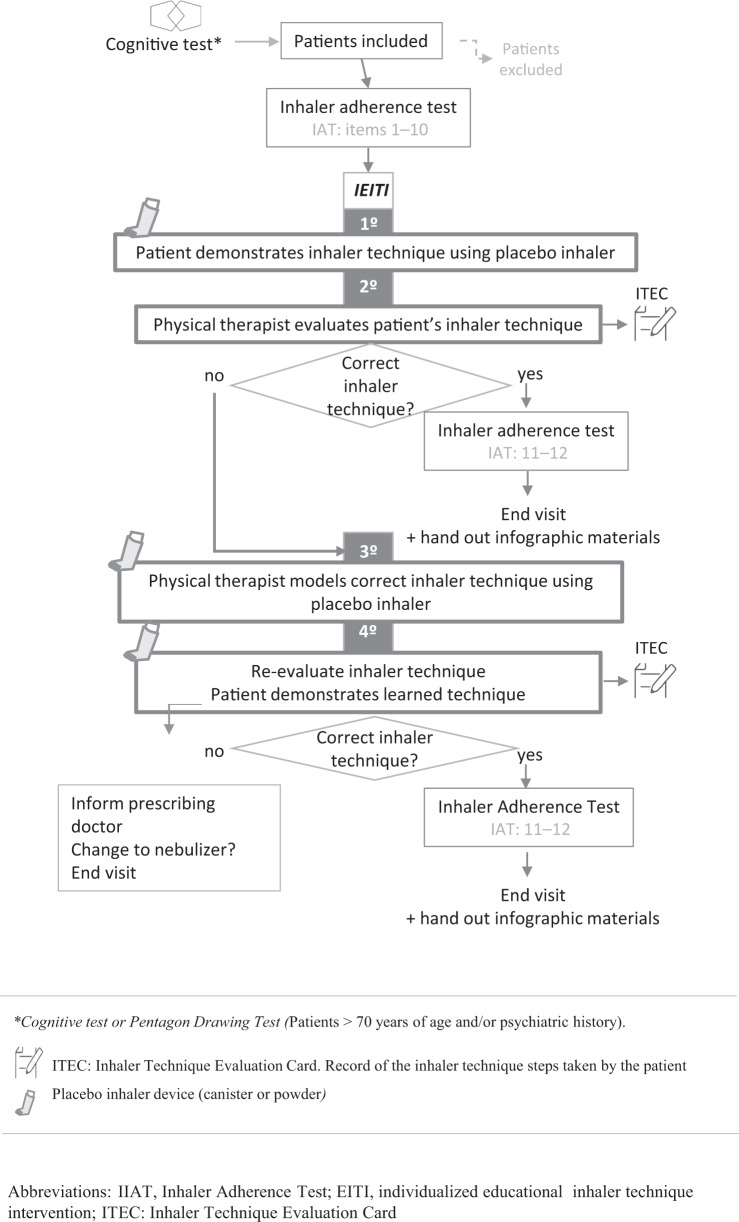
Individualized educational inhaler technique intervention (IEITI): four stages.

Following the recruitment phase, they were scheduled for an initial visit (IV) with a physical therapist who was not involved in the recruitment. Two Pulmonology investigators recruited patients in the consultation. The Physical therapists were trained during several sessions until they master the competence in inhalation technique training and using the TAI test. During this visit, IEITI was done and a final visit (FV) was performed on week 24. The IEITI consisted of an educational intervention based on the teach-bak model^23^. The patient received verbal instruction on the inhaler technique and then was asked to show their ability to do it. When the patient does not show an acceptable skill technique, further instructions are given until he achieved that. The patient did not show an acceptable level of skill if, after explanations followed by physiotherapy and two consecutive patient demonstrations, he could not perform the loading of the system and/or the inspiratory maneuver. The sequence of study visits is shown in Fig. 1 and the systematic training, divided into four consecutive stages, is explained in Fig. 2. The IEITI also included informational material on dosage, scheduling, and characteristics of the inhalers (Supplementary Fig. 1).

### Data collection

The degree of adherence to the inhaler treatment was evaluated using the ten-item TAI^15^ (https://www.taitest.com/). Each item scored from 1 to 5 (where 1 was the worst possible score and 5 was the best possible score), resulting in a minimum score of 10 points and a maximum of 50 points. Three levels of adherence were established along this continuum: poor (≤45), intermediate (46–49), and good (50). For this study, we have used a composite variable that we have named Low Adherence (LowAd), which includes all patients with “intermediate” and “poor” adherence, according to the cut-off points established by the authors, with the purpose to facilitate the interpretation of the results. Therefore, LowAd patients are those with a score ≤49. Consequently, patients with Optimal Adherence (OptAd) are those with a score of 50 points. The “complete TAI” includes two additional questions (12-item TAI^15^), performed asked by the professional in order to explore nonadherence or noncompliance patterns. In item 11, 1 point is given if patients do not remember the dosage or frequency, and 2 points are given if they remember it. In item 12, 1 point indicates that the patient makes some critical error in inhaler technique whereas 2 points indicate that the patient does not make any errors when using the inhaler. Three patterns of noncompliance have been identified by dividing up the scores into three groups of questions: “erratic” <25 points (items 1–5), “deliberate” <25 points (items 6–10), and “unwitting” <4 points (items 11–12).

### Individualized educational inhaler technique intervention (IEITI)

**Fig. 3:**
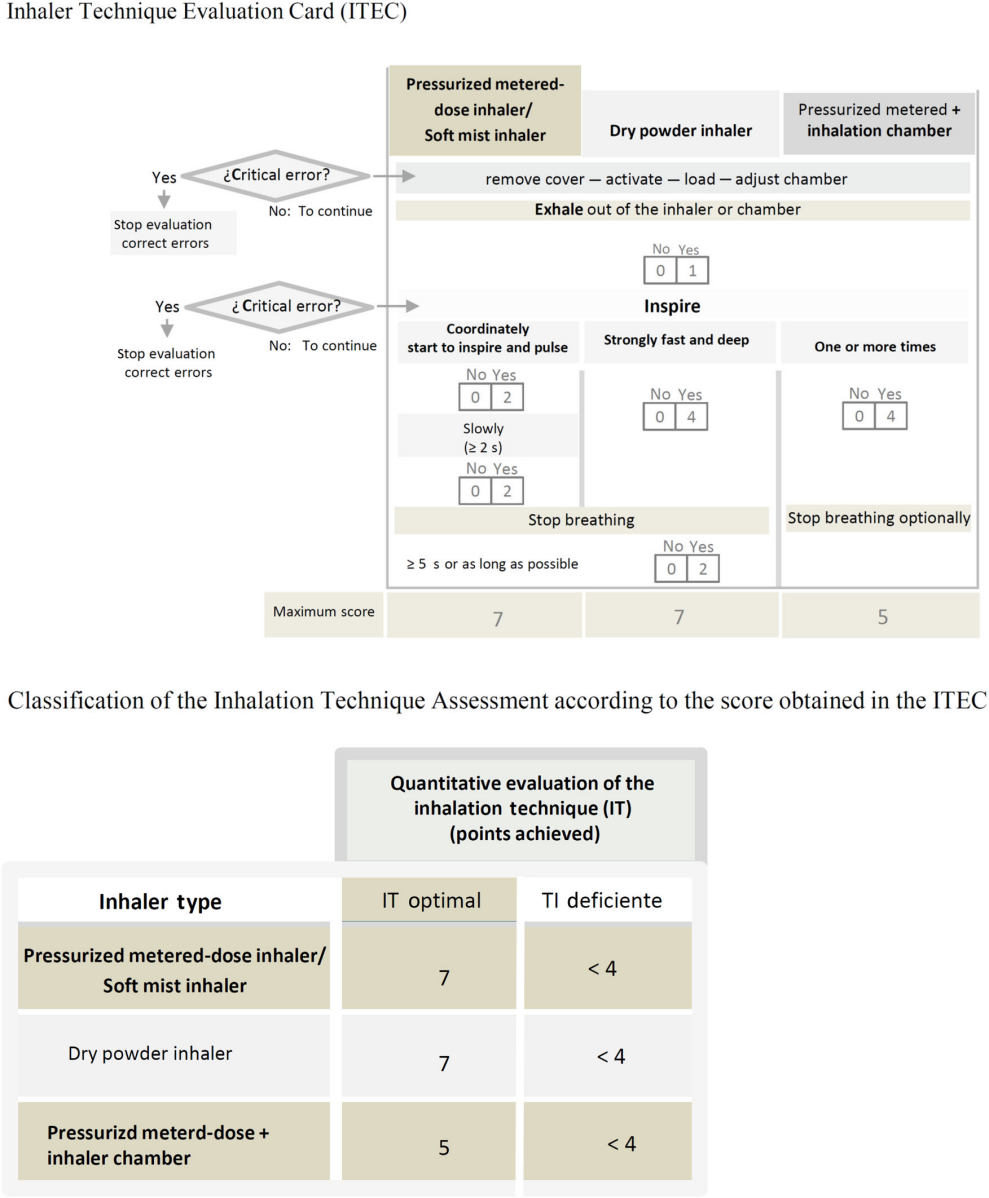
Inhaler technique evaluation card (ITEC).

The stages of IEITI are shown in Fig. 2. An IEITI was carried out in an individual session of 30–40 min, conducted by a physical therapist. The session included the demonstration and assessment of the inhaler technique. Errors were corrected until the patient reached an acceptable technique. Prior to the intervention, the patient was asked to complete the TAI (10 items). At the beginning of the session, the therapist asks the patient to show how he uses the inhaler prescribed, before receiving any instructions for correct use. For that purpose, the patient received an identical device with a placebo. It was considered that the patient had a Deficient Inhaler Technique (DeIT) when the inspiratory flow maneuver was insufficient and/or a critical error was made. The results of the evaluation were entered on an inhaler technique evaluation card (ITEC) (Fig. 3). Next, the patient was asked about the dosage and frequency of the inhaler (item 11). If they have not made any “critical error^24^” (item 12), a graphic material of the inhaled medication is given (Supplementary Fig. 1) and the session is concluded. “Critical error” were considered if the patient showed an action or inaction that, in itself, which can lead to a detrimental impact on drug administration in the lung^24^. If the patient does not present an acceptable skill level, the physical therapist will proceed to the correct inhaler use model, correct errors, and ask the patient to show what he learned through this process. When the patient continued displaying DeIT, the therapist contacted the prescribing doctor to report that the inhaler needed to be changed.

### Clinical variables and outcome measure

Data was gathered on sociodemographic, clinical, and spirometric information, type of inhaler evaluated, and the results of the inhaler technique skill level of the patient (optimal or poor inhaler technique). The primary and secondary variables were analysed at the baseline and after the intervention (IEITI). The main variable was the decrease in the percentage of patients with LowAd in the final visit. Secondary variables were: types of noncompliance: erratic unwitting and deliberate, and percentage of patients with poor Inhaler Technique and critical errors. The differences between patients diagnosed with asthma and COPD were also analysed as part of the study variables. To minimize measurement bias, the evaluation of the last visit, in week 24, was performed by a nurse previously trained in conducting the questionnaire TAI, blinded to the results of the initial questionnaire and who has not participated in the initial training.

### Statistical analysis

The sample size was calculated in bilateral contrast factoring in a 5% alpha risk and 0.1 beta risk (90% statistical power). A sample of 130 participants is necessary assuming that the initial rate of LAd would be 45% and the final rate 25%^25–27^. The rate of patient loss to follow-up was estimated at 8%. Quantitative variables are shown as averages ± standard deviation (interquartile range: first and third quartile). Comparisons between groups were performed with the Fisher exact test. Categorical variables were expressed as absolute and relative frequencies, and comparisons between them were made using the Pearson Chi^2^ test or Fisher’s test. Quantitative variables were expressed as mean ± standard deviation and the comparisons were made between independent groups using the Student’s *t*-test or the Mann–Whitney test if the variable did not present a normal distribution. When the variables have been measured at different points, the McNemar test was used for their comparison and the paired samples *t*-test or Wilcoxon test depending on whether or not the distribution of the quantitative variables.

A multivariate logistic regression analysis was performed to evaluate associated factors with LowAd, calculating the odds ratios (ORs) and 95% confidence intervals (CI). The independent variables considered were: age, sex, deficient inhaler technique (initial visit), smokers status, previous training, the severity level of disease (COPD/Asthma), type of disease (COPD/asthma), and types of inhalers evaluated (initial visit). First, a univariate analysis of each variable was performed, and then, the variables whose univariate test had a *p* value <0.3 were included in the multivariate logistic regression model. The goodness-of-fit of the multivariate model was evaluated with the Hosmer–Lemeshow test. Odds ratio (OR) values were calculated with 95% confidence intervals (CI 95% CI). All analyses were performed “two tails”, and a *p* value of less than 0.05 was considered significant. All analyses were performed with the SPSS statistical software program (SPSS version 25.0; IBM®, Armonk, NY) and Stata [StataCorp. 2015. Stata Statistical Software: Release 14. College Station, TX: StataCorp LP]. A blinded researcher carried out the data analysis.

### Reporting summary

Further information on research design is available in the Nature Research Reporting Summary linked to this article.

## Results

### Participants

**Fig. 4:**
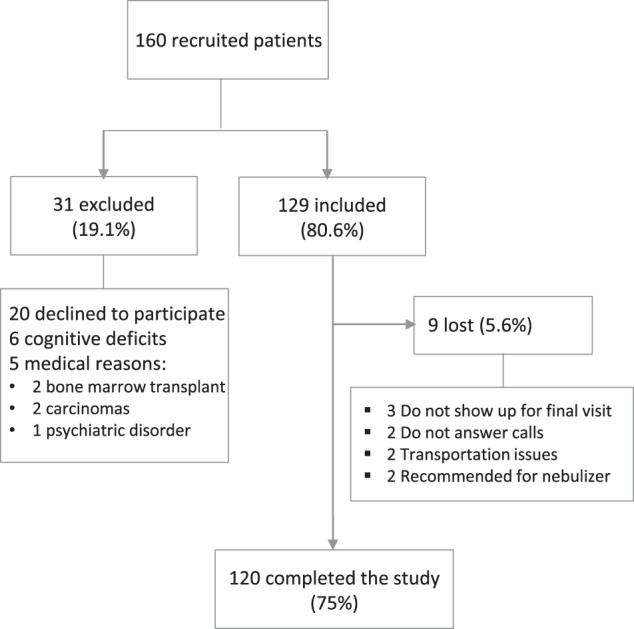
Study inclusion and follow-up algorithm.

A group of 160 patients was recruited for the study. Of these, 31 (19.1%) were excluded, a majority of which, 20 (64.5%), refused to attend the visits. Nine patients (5.6%) were lost to follow-up. Two patients have prescribed a home nebulizer due to repeated critical errors in inhaler technique and120 finished the study. The inclusion criteria and follow-up algorithm are shown in Fig. 4.

Most of the patients were female (55.8%) with an average age of 60,8 (±16.6) years. Most of them had been diagnosed with asthma, 85 patients (70.8%) and the rest, 35 patients, were diagnosed with COPD. The average FEV_1_ was 72.6 ± 20.4 (of the predicted value). 45% of the patients report having some previous training in the use of the inhaler that was prescribed. The remaining baseline characteristics of the 120 patients that participated in the study are shown in Table 1.

**Table 1:** Baseline characteristics of patients in the initial visit (*n* = 120).

Characteristic		*N* = 120	%
Female, *n* (%)		67	55,8
Age, years, mean ± SD		60,8 ± 16.6	
Asthma diagnosis	Mild-intermittent	54	63,8
	Moderate-severe	31	36,5
COPD Diagnosis	Mild-moderate	20	57,1
	Severe-very severe	15	52,9
Smokers status, *n* (%)		25	20,8
Previous training*, *n* (%)		54	45
Number drugs /patient**, mean ± SD		2 (1,5)	
Number inhalers/patient***, mean ± SD		2 (2,2)	
No studies or primary, *n* (%)		83	69,2

*SD* standard deviation, *FEV1* forced expiratory volume in 1 second, *COPD* chronic obstructive pulmonary disease.

*some form of “unstructured” instruction, **excluded inhalers, ***were evaluated 430 inhalers.

The average score in the ten-item TAI questionnaire was 43,1 (±8,8) points in the initial visit and 46.6 (±5.9) at the end of the study (*p* < 0.001). About 120 inhalers were evaluated in the initial and final visits. The most commonly used inhaler at study recruitment was multidose DPIm, in 52 patients (43.3%) followed by pMDI with spacer chamber, in 31 patients (25.8%). The numbers and types of inhalers evaluated in the visits are listed in Table 2.

**Table 2:** Types of inhalers evaluated in the study visits.

Type inhaler	Initial visit	Final visit
	*N* = 120 (%)	*N* = 120 (%)
pMDI	12 (10)	18 (15)
pMDI + chamber spacer	31 (25,8)	24 (20)
Soft mist inhaler	12 (10)	19 (15,8)
MDPI	52 (43,3)	48 (40)
UDPI	13 (10,8)	11 (9,1)

*pMDI* metered-dose inhaler pressurized, *MDPI* inhaler dry powder multidose, *UDPI* inhaler dry powder unidose.

Based on the definition established in this study to evaluate inhaler technique, during the IV it was determined that the technique was poor or deficient in 69 inhalers (72.8%) and a critical error was made in the manipulation of 21 inhalers (16.3%). Regarding the level of adherence, during the IV, 81 patients (67.5%) had LowAd. The most frequent form of noncompliance was forgetting to use the inhaler in 65.8% of the patients (Noncompliance erratic). Lack of knowledge of the dosage and/or inhaler technique (unwitting), was the second most common form of noncompliance, in 69 patients (57.5%). Finally, nonadherence that is deliberate and largely associated with patient motivation to use the inhaler, was identified in 52 patients (43.3%).

### Effects of the intervention on the study variables

During the IV, 81 patients (67.5%) presented LowAd compared to 66 (55%) in the FV. In contrast, the number of patients that presented OptAd at the start, 39 (32.5%), had increased to 54 (45%) at the end of the study. The intervention (IEITI) produced a significant change in the level of adherence (*p* = 0.024) and a decrease in the rate of patients with LowAd on week 24 of the study. There was a decrease in erratic, 79 patients (65.8%), in the IV vs 62 (51.7%) after the IEITI (*P* = 0.009). The number of patients presenting noncompliance deliberate went from 52 (43.3%) to 31 (25.8%) at the end (*p* = 0.002). Lastly, out of 69 patients (57.5%) with unwitting noncompliance, 47 (39.2%) remained in this category at the end (*p* = 0.002). The pattern and relative frequency of noncompliance did not change by the end of the study, being the erratic pattern the most common one. Regarding the secondary variables, a significant change was found in the percentage of inhalers that were used with poor inhaler technique. Similarly, the percentage of critical errors found in the initial visit improved after the IEITI. Table 3 shows the description of the level of adherence, noncompliance, technique, and critical errors in the initial and final visit.

**Table 3:** Changes in adherence, type of noncompliance and critical errors in initial an final visit.

	Initial visit	Final visit	*P*
Adherence (items 1–10 TAI)	*n* (%)	*n* (%)	
Low adherence (≤49 points)	81 (67,5)	66 (55)	0.024
Optimal adherence (=50 points)	39 (32,5)	54 (45)	
Types noncompliance (items 1–12 TAI)			
Erratic noncompliance (items 1–5)	79 (65,8)	62 (51,7%)	0.009
Ignorant noncompliance (items 11–12)	69 (57,5)	47 (39,2%)	0.002
Deliberate noncompliance (items 6–10)	52 (43,3)	31 (25,8%)	0.002
Critical errors (Item 12 TAI)	21 (16,3%)	3 (2,5%)	0.461
Evaluations with poor inhalation technique*	69 (57,5%)	22 (11,2%)	0.002

*TAI* test of the adherence to inhalers, *DeIT* deficient inhaler technique.

*Deficient inhaler technique (DeIT).

**Fig. 5:**
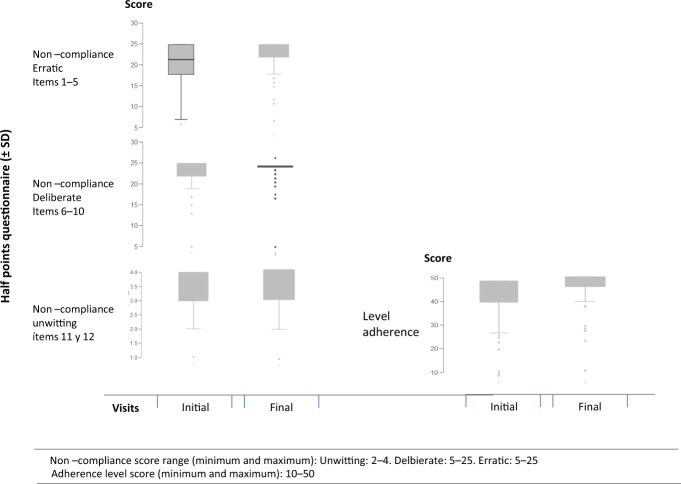
Average scores (±SD) were obtained by the patients according to the established ranges to classify the type of noncompliance and the level of Adherence.

Figure 5 shows point averages ± standard deviation of the patients in the initial and final visit according to the cut-off points established for the classification of noncompliance and level of adherence

No significant differences were identified between the group of patients with COPD and asthma. Patients with asthma presented a higher rate of LowAd than patients with COPD, 71.8 vs 57.1% (*p* = 0.120). Deliberate noncompliance was also most frequent in patients with asthma, 48 vs 31.4%. In contrast, asthma patients displayed a better skill level in the use of the inhalers. 52.9% asthma patients had DeIT vs 68.6% in COPD patients (*p* = 0.039). The differences between patients with COPD and asthma are shown in Table 4.

**Table 4:** Changes in adherence, type of noncompliance, and critical errors between COPD and asthma.

	COPD	ASTHMA	
	*n* (%)	*n* (%)	*P*
Adherence (items 1–10 TAI)			
Low adherence (≤49 points)	20 (57,1)	61 (71,8)	0.120
Types noncompliance (items 1–12 TAI)			
Erratic noncompliance (items 1–5)	20 (57,1)	59 (69,4)	0.198
Unwitting noncompliance (items 11–12)	20 (57,1)	49 (57,5)	0.095
Deliberate noncompliance (items 6–10)	11 (31,4)	41 (48,2)	0.091
Critical errors (item 12 TAI)	4 (11,4)	14 (16,5)	0.482
Inhalation technique*	24 (68,6)	45 (52,9)	0.115
Evaluations with por			
inhalation technique*	24 (68,6)	45 (52,9)	0.115

*TAI* test of the adherence to inhalers, *COPD* chronic obstructive pulmonary disease, *DeIT* deficient inhaler technique.

*Deficient inhaler technique (DeIT).

The baseline characteristics of the patients, such as having received previous training or their level of studies, showed no relationship with low adherence. Only gender was related to low adherence (Table 5). Finally, age, gender, and asthma diagnosis were chosen for the multivariate adjustment. The analysis did not show any relation between the level of adherence and baseline characteristics of the patients except in the case of being a female patient (OR = 2.384, IC: 1.039–5.5518; *p* = 0.040). (Table 6).

**Table 5:** Relation of the low and optimal adherence and baseline characteristics of the patients with COPD and asthma.

Characteristics		Low adherence	Optimal adherence	*P*
		45 (69.7)	22 (38.9)	0.001
Females		58.9 ± 16.7		
		80.7 ± 23.7	70.9 ± 20.1	0.073
Asthma diagnosis	Mild-intermittent	31 (62)	23 (65.7)	*0.726
	Moderate-severe	19 (38)	12 (34.3)	
COPD diagnosis	Mild-moderate	9 (56.3)	11(57.9)	*0.922
	Severe-very severe	7 (43.8)	8 (42.1)	
Smoker		16 (24.2)	9 (16.7)	0.429
Previous inhaler technique training **		30 (45.5)	24 (44.4)	0.912
Medications /patient excluding inhalers		2 ± 1.5	2 (0.5)	0.746
Inhalers/patient		2 ± 2.2	2 (0.5)	0.456
No formal or basic education		44 (66.7)	39 (72.2)	0.512

The dates show *n* (%) or average ± standard deviation.

*COPD* chronic obstructive pulmonary disease.

*P result severity of asthma and COPD, ** some form of “unstructured” instruction.

**Table 6:** Logistic regression model.

Variable	OR	IC-95% OR	*p* value
Age >70	0.591	0.252–1.394	0.231
Female	2.394	1.039–5.518	0.04
Asthma diagnosis	1.384	0.573–3.341	0.469

The variables identified as predictors of low adherence on univariate analysis: age, gender and asthma or COPD diagnosis, were included. For this model, the Hosmer–Lemeshow test showed a *p* value = 0.699.

*OR* odds ratio, *IC* interval confidence, *COPD* chronic obstructive pulmonary disease.

## Discussion

The problem of low adherence to inhaled treatment of chronic respiratory disease includes numerous factors of different nature and complexity. The perception of a therapeutic benefit by the patient and the effective use of the inhaler are the key to achieve adherence to the treatment. Insufficient instructions on the use of the inhaler and poor inhaler technique are common and have negative repercussions on adherence in the case of asthma and COPD^8,9^. Several studies have evaluated the implementation of interventions to improve inhaled treatment adherence. Interventions vary from providing only information in different formats to complex self-management programs and have had uneven results^8^. Nine authors used “teach-back*”* interventions similar to those used in the present study and evaluated their impact on the proportion of patients with the correct use of the inhaler but did not look at the changes in the adherence^23^. Other reviews have evaluated multi-component strategies to improve adherence but it is hard to determine the contribution of each component to the outcomes. It is also difficult to compare the results due to the diversity of methods employed to evaluate adherence^8^. An observational study with 88 patients with COPD that evaluated adherence by means of a four-question *self-administered questionnaire* found that the only factor significantly related to adherence was having received instructions of inhaler technique previously^28^.

We did not find any studies on the impact of adherence of an inhaler technique education intervention, using the IAT, on a population with asthma and COPD. After the IEITI intervention, patients with LowAd decreased significantly and at the same time, patients with optimal adherence increased. The types of noncompliance, the percentage of patients with poor inhaler technique, and the percentage of critical errors also improved. A recent metanalysis addressed the impact of these interventions on asthma and COPD exacerbations^29^. Only three studies evaluated the impact on adherence to the inhaled medication although, according to the authors, the benefit could be explained, in part, through the so-called Hawthorne effect: the awareness of being observed or of having a behavior that is being evaluated, generates beliefs about the researcher’s expectations and considerations of social acceptance that lead to a change in behavior^30^.

Also, different measures of adherence were used and, finally, these were not included in the quantitative analysis.

The percentage of LowAd in the COPD and asthma population in the study is similar to those reported by other authors who used different measurement instruments^7^. About 67.5% of the patients in our study presented a low level of adherence, with an average score of 43.1 ± 8.8 (10-item TAI). The TAI^15^ and other recent observational studies report similar results^31–33^. The first, which was carried out among Asian patients with exacerbated COPD, reported low adherence in 70% of the cases (low + intermediate adherence)^32^. Another study which was carried in Spain with 122 COPD and asthma patients found low adherence in 71.3% of the patients studied^32^. However, a multinational study conducted in Latin America with 795 patients found surprisingly good adherence results. The average score was 47.4 ± 4.9 and the percentage of LowAd in this population was 45.9%^33^.

When we analyze separately the levels of adherence in patients with asthma and COPD, we found a LowAd level in asthma patients (57.1%) compared with COPD patients (71.8%), although without any significant differences. A multicentric study that analysed these differences using the same TAI instrument, found significant differences in levels of adherence in both groups of patients, with a higher rate of LowAd in asthma patients (72%) and lower in COPD patients (51%)^34^. The noncompliance patterns between COPD and asthma are also different in this study, being the most frequent pattern in asthmatics the erratic (66.8%). These differences are more likely to be related to sociodemographic characteristics^34^. In our study, the erratic pattern was also higher in patients with asthma with very similar values (69%). In this group of patients, at baseline, the frequency of the erratic pattern was 65% compared to 57.9% that was obtained in the validation work of the TAI^15^. These studies^15,34^ did not include educational interventions nor a longitudinal evolution analysis of patient adherence.

In relation to the evaluation of the inhalation technique, there is high variability in the comparison of results due to the heterogeneity of the methods used. In general, the ability of patients in the inhalation technique seems not to have improved in the last 40 years^35^. The international study “*International Helping Asthma in Real-life Patients*” (iHARP), the largest asthma study on patient inhaler technique with 5000 structured evaluations, showed an error rate for inhalers (pMDI and DPIm) higher than 90% ^36^. At this point, we must comment on our results. Unlike other studies^35^, our definition of poor inhalation technique was not based on a strict recording of an error checklist. Only the presence of an insufficient or uncoordinated inspiratory step and/or the existence of a critical error led us to consider an inhalation technique as deficient. Following the opinion of some authors^37^, some steps such as exhaling before inhaling and/or the absence of apnea were not considered sufficient to consider the inhalation technique as deficient. These considerations may represent a lower percentage of DeIT than reported in other studies^36^. Something similar happens with the disparity of assessments of inhaler technique critical errors^24^.

Our results suggest that educational interventions on inhaler techniques improve patients’ ability and, at the same time, can also improve the perception of therapeutic benefit and adherence to inhaled medication. Although, these results should be interpreted with caution. First, the efficacy of a healthcare intervention is ideally demonstrated under the conditions of double-blind randomized controlled trials with highly selected populations and operating under highly monitored and controlled conditions^38^. However, logistical limitations conditioned the design to a pre-post intervention study, thus incorporating possible biases to the results obtained. Occasionally, studies with minimal exclusion criteria may be more representative of the patients seen in daily clinical practice and provide complementary data to those obtained in traditional efficacy studies^39^.

Second, it is possible that the modifications in the patients’ behavior could have influenced the results of the IAT in the final visit since the patients knew that they were being evaluated and not as an effect of the intervention itself (Hawthorne effect)^30^. Having a wide age range in the study may have introduced a bias, mainly due to endotypic and phenotypic differences. This could have led to different clinical and questionnaire responses to the educational intervention^27^. Another aspect that should be considered when interpreting the results is the measure of adherence to the inhaled medication by means of a self-administered questionnaire due to the biases inherent to this type of qualitative instrument^14^. Recent studies show evidence of an overstatement of adherence in patients evaluated using the TAI compared to medication administration records^40,41^. The TAI seems to be more reliable when assessing patients with low adherence. But, with higher scores, it should be modulated with more objective methods, particularly in the context of studies of intervention effectiveness^40^. In our study, the TAI was evaluated longitudinally in two visits. This bias could have been present in both measurements, but it did not condition the favorable evolution of adherence in a significant way. Another limitation of the study is the sample size, which was slightly lower than the calculated sample size, and the possible impact of other unmeasured confounding or covariates not included in the variable selection in the logistic regression model, such as the educational or socioeconomic level of the patients. Finally, the possibility of a regression problem of the mean, although this phenomenon is frequent when there is a change between two measurements, where the first shows a value and the second is closer to the mean. Despite the overall improvement of patients in the score of the TAI questionnaire is slight, it rises three points on average in the second measurement, we believe that this is not the main finding of the study, but rather a 12% improvement in optimal adherence.

Given the substantial cost of asthma and COPD management, it is necessary to continue developing strategies to optimize the benefits of inhaled medication. There are still many aspects that need to be researched in relation to adherence and inhaler technique skill, particularly in “real-life” studies. Among the future needs pointed out by authors like Price et al.^42^, is needed a more holistic healthcare system, with an integrated approach to optimize the inhaled treatment and adherence. To achieve this objective, it is necessary to better understand the conceptual connection between adherence and technique (whether they are different aspects, or they must be combined into one integrated quality approach to the administration of inhaled medication). Understanding behavioral patterns of adherence in a subpopulation of patients (e.g., children, adults) and at different stages of the disease, will help to develop more specific and effective interventions. This study can contribute to the understanding of how adherence and inhaler technique interact by evaluating them longitudinally following a structured educational intervention in real-world clinical practice conditions.

We demonstrated that, among patients with COPD and asthma, an individualized educational inhaler technique intervention, carried out in real-world clinical practice conditions, improves adherence to the inhaled treatment, as evaluated by means of TAI. However, the small sample size limits the external validity of these results and suggests the need for further studies.

## Supplementary information

Supplementary Information

Reporting Summary

## Data Availability

The data that support the findings of this study are available from the corresponding author upon reasonable request. The original contributions presented in the study are included in the article/supplementary material (Supplementary Fig. 1). Correspondence and requests for supplementary materials should be addressed to the corresponding author.
