# Supplementary material for: Effectiveness of individualized inhaler technique training on low adherence (LowAd) in ambulatory patients with COPD and asthma
Source: NPJ Prim Care Respir Med. 2022 Jan 10;32:1. doi: 10.1038/s41533-021-00262-8 (PMC8748930; doi:10.1038/s41533-021-00262-8)
Supplement: Supplementary file 1 — Supplementary Information [file 41533_2021_262_MOESM1_ESM.pdf]

Appendix I

Informational material on dosage, scheduling and characteristics of the inhalers

Use Guide Inhalers

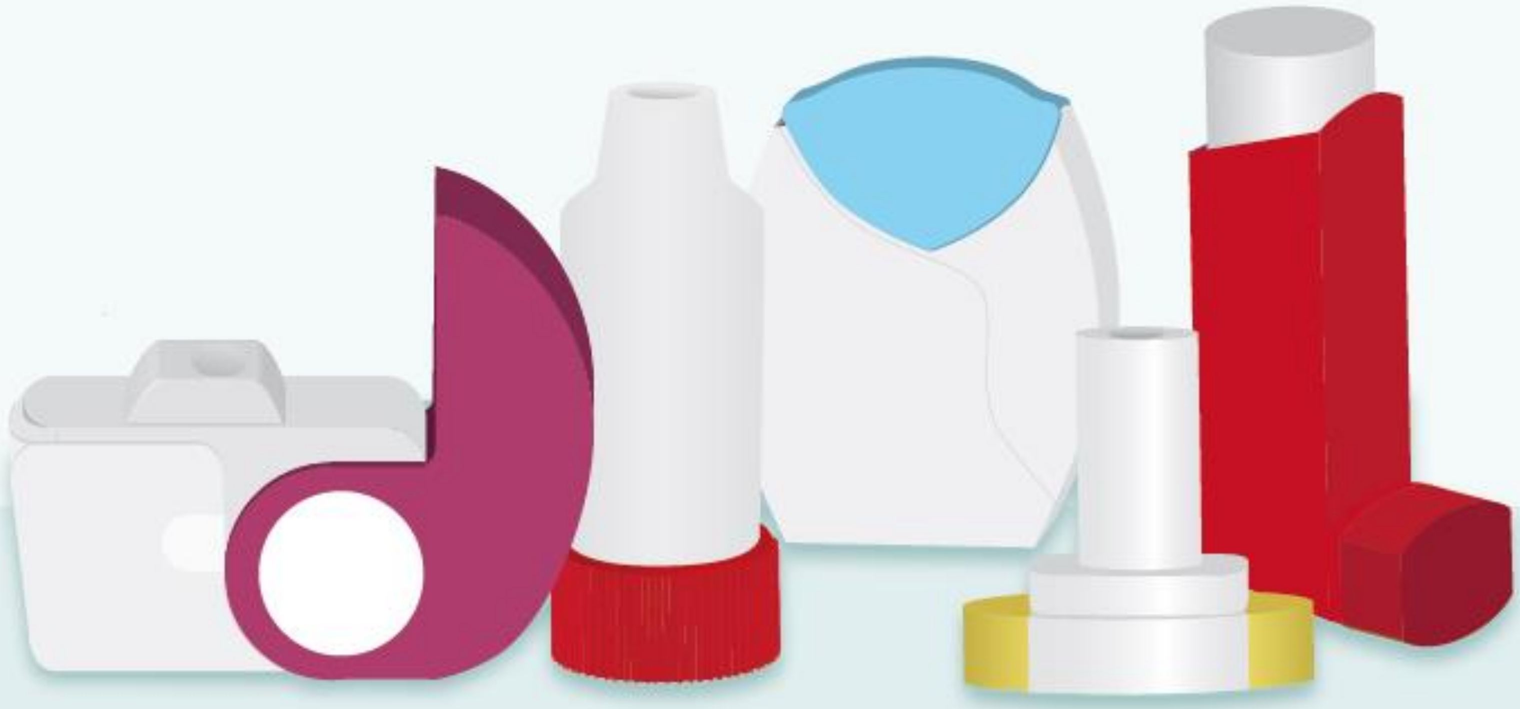

Copyright© SÁNCHEZ NIETO, JUAN MIGUEL, 2017. RPTI N° 08/2017/168

Advices and general information

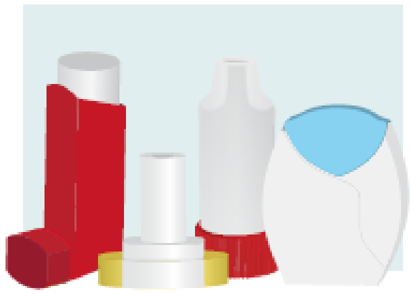

The best way to take medicines is like inhalers taking advantage of the breath

Taking inhalers has advantages. They can be taken before and after eating

It is advisable to rinse the mouth after taking them

Use inhalers at the same time. In case of forgetfulness, wait for the next dose

They should be stored in a dry place

The *inhalation chamber* makes it easier to take some inhalers and increases the amount of medicine reaching the bronchial tubes

"Rescue inhalers" can be taken without a set schedule, when the patient feels short of breath or before exertion

Copyright© SÁNCHEZ NIETO, JUAN MIGUEL, 2017. RPTI Nº 08/2017/168

Pressured metered dose inhaler

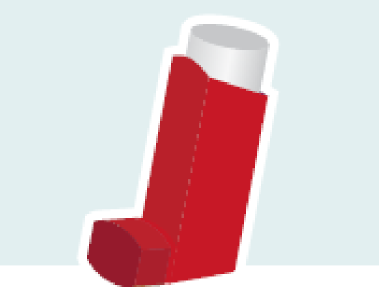

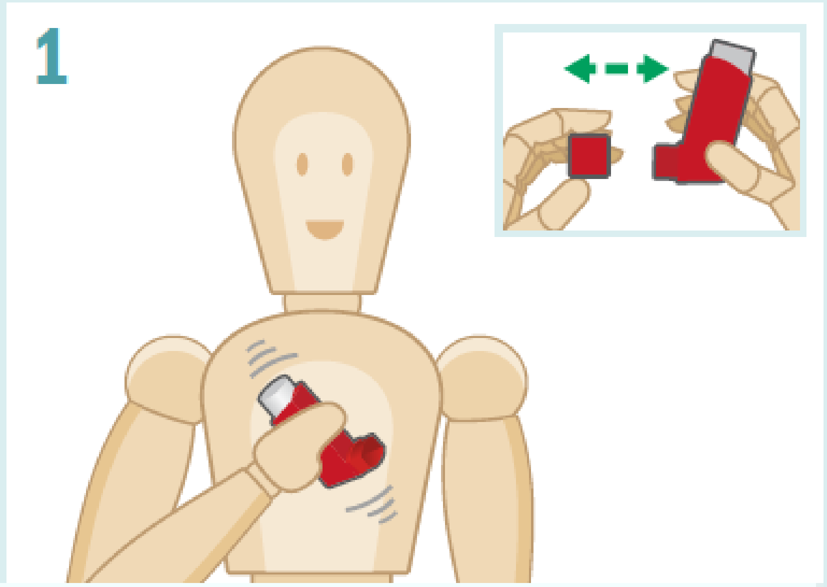

Remove the cap and shake the inhaler

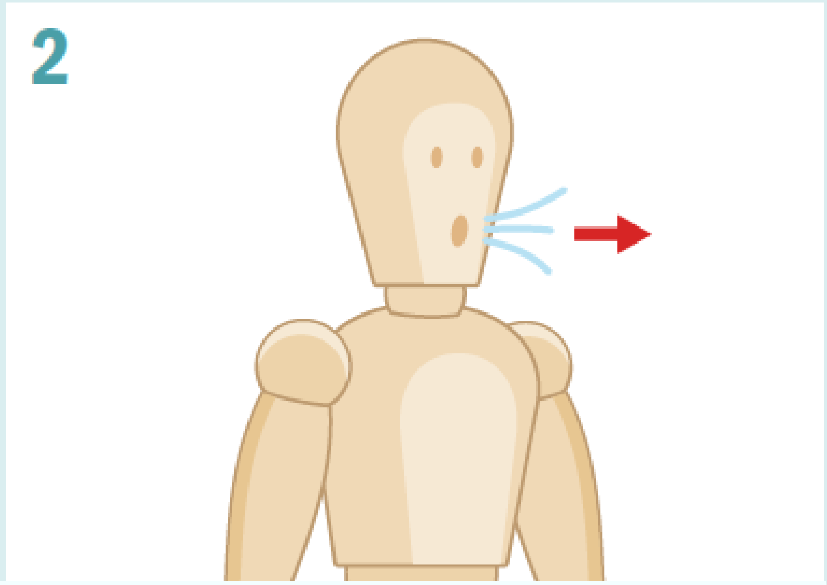

Breath out until your chest is empty

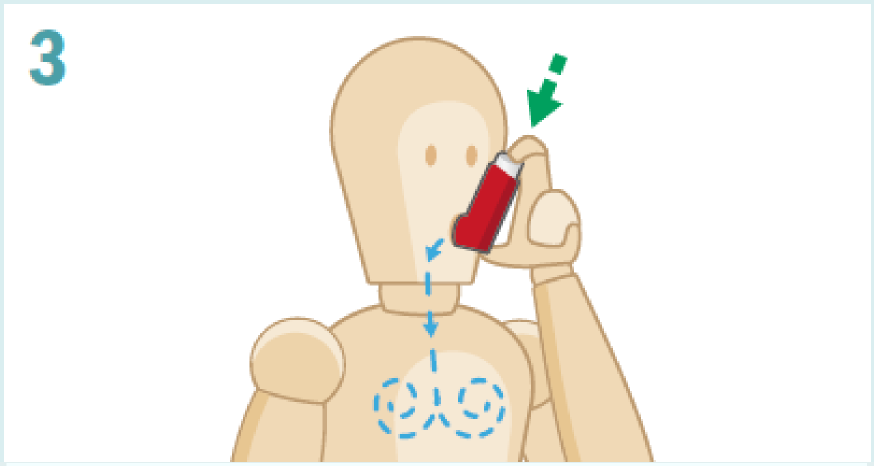

Press the inhaler just when you start to take in air and continue to breathe slowly

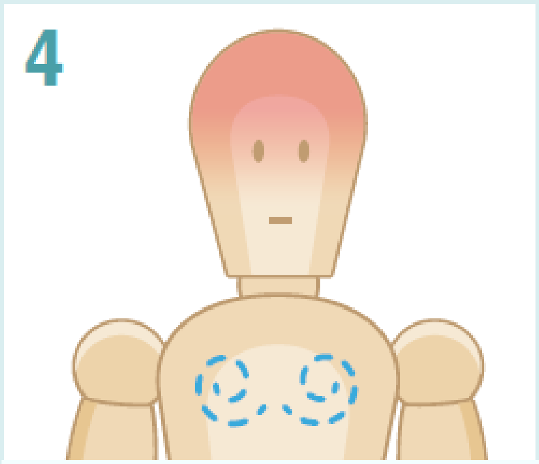

keep without breathing as much as possible (3 to 5 seconds)

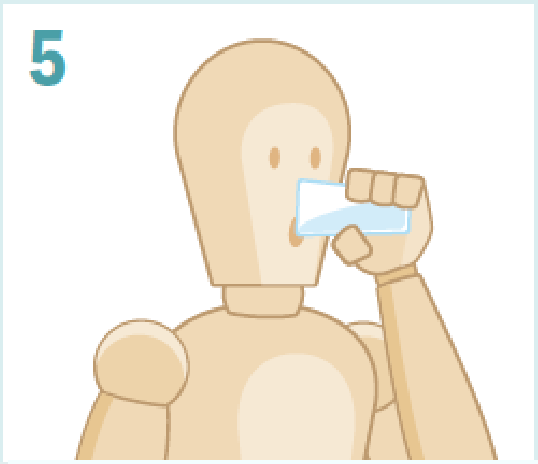

Rinse by mouth with water, without swallowing

Copyright© SÁNCHEZ NIETO, JUAN MIGUEL, 2017. RPTI Nº 08/2017/168

Multi-dose powder inhalers: Turbuhaler®

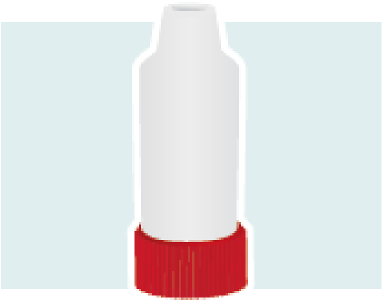

1

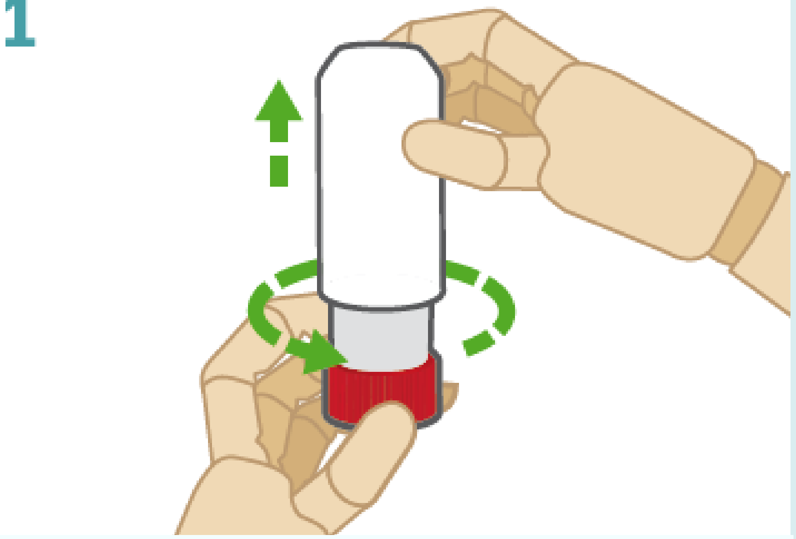

Unscrew and remove the cap

2

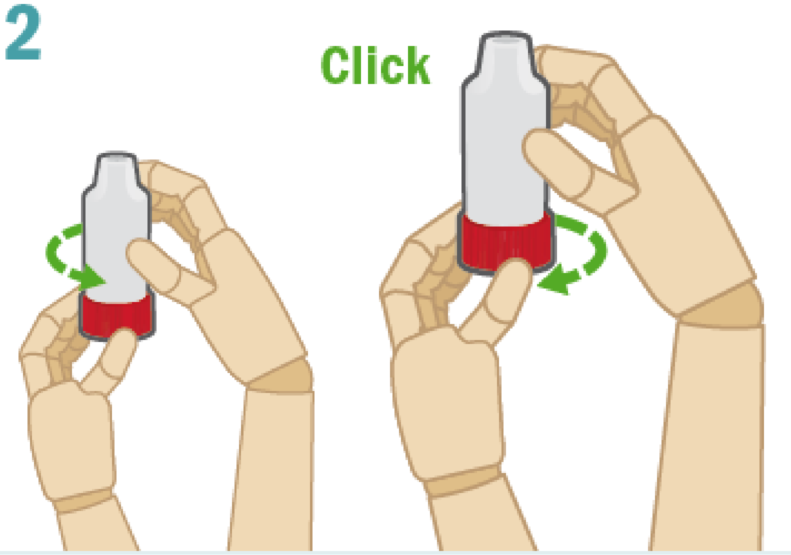

Spin the wheel until you hear it click!

3

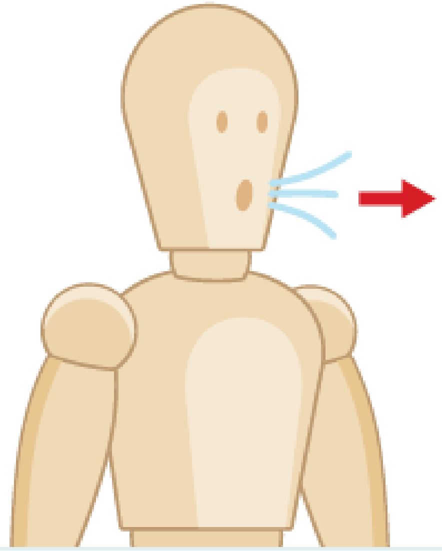

Breath out until your chest is empty

4

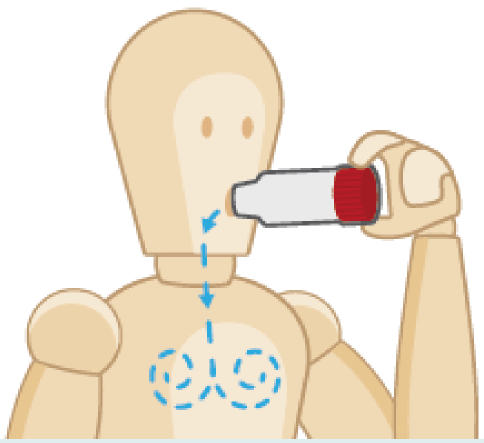

Breathe in forcefully, as fast and deep as possible

5

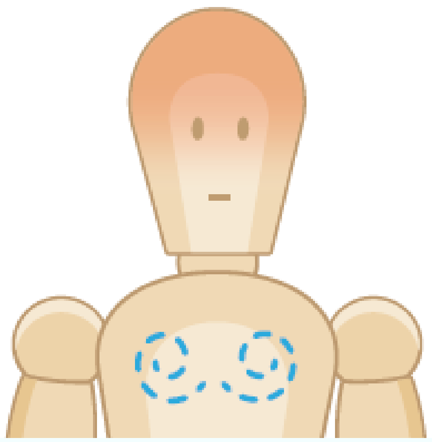

keep without breathing as much as possible
(3 to 5 seconds)

6

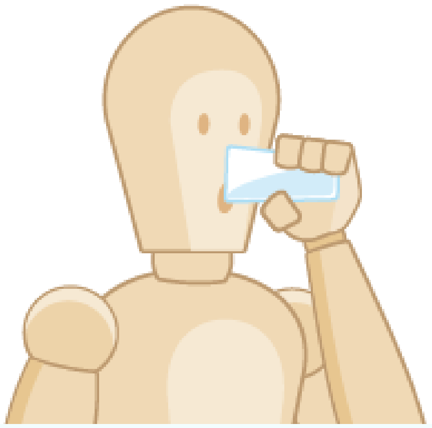

Rinse by mouth with water, without swallowing

Copyright© SÁNCHEZ NIETO, JUAN MIGUEL, 2017. RPTI Nº 08/2017/168
