# Supplementary material for: Effectiveness of individualized inhaler technique training on low adherence (LowAd) in ambulatory patients with COPD and asthma
Source: NPJ Prim Care Respir Med. 2022 Jan 10;32:1. doi: 10.1038/s41533-021-00262-8 (PMC8748930; doi:10.1038/s41533-021-00262-8)
Supplement: Supplementary file 2 — Reporting Summary [file 41533_2021_262_MOESM2_ESM.pdf]

nature portfolio

Corresponding author(s): RBM

Last updated by author(s): Jun 29, 2021

Reporting Summary

Nature Portfolio wishes to improve the reproducibility of the work that we publish. This form provides structure for consistency and transparency in reporting. For further information on Nature Portfolio policies, see our Editorial Policies and the Editorial Policy Checklist.

Statistics

For all statistical analyses, confirm that the following items are present in the figure legend, table legend, main text, or Methods section.

n/a	Confirmed	
☐	☒	The exact sample size (n) for each experimental group/condition, given as a discrete number and unit of measurement
☐	☒	A statement on whether measurements were taken from distinct samples or whether the same sample was measured repeatedly
☐	☒	The statistical test(s) used AND whether they are one- or two-sided<br>*Only common tests should be described solely by name; describe more complex techniques in the Methods section.*
☐	☒	A description of all covariates tested
☐	☒	A description of any assumptions or corrections, such as tests of normality and adjustment for multiple comparisons
☐	☒	A full description of the statistical parameters including central tendency (e.g. means) or other basic estimates (e.g. regression coefficient) AND variation (e.g. standard deviation) or associated estimates of uncertainty (e.g. confidence intervals)
☐	☒	For null hypothesis testing, the test statistic (e.g. F, t, r) with confidence intervals, effect sizes, degrees of freedom and P value noted<br>*Give P values as exact values whenever suitable.*
☒	☐	For Bayesian analysis, information on the choice of priors and Markov chain Monte Carlo settings
☐	☒	For hierarchical and complex designs, identification of the appropriate level for tests and full reporting of outcomes
☒	☐	Estimates of effect sizes (e.g. Cohen's d, Pearson's r), indicating how they were calculated

Our web collection on statistics for biologists contains articles on many of the points above.

Software and code

Policy information about availability of computer code

Data collection	No sofware was used
Data analysis	All analyses were performed with the SPSS statistical software program (SPSS version 25.0; IBM®, Armonk, NY) and Stata [StataCorp. 2015. Stata Statistical Software: Release 14. College Station, TX: StataCorp LP].

For manuscripts utilizing custom algorithms or software that are central to the research but not yet described in published literature, software must be made available to editors and reviewers. We strongly encourage code deposition in a community repository (e.g. GitHub). See the Nature Portfolio guidelines for submitting code & software for further information.

Data

Policy information about availability of data

All manuscripts must include a data availability statement. This statement should provide the following information, where applicable:
- Accession codes, unique identifiers, or web links for publicly available datasets
- A description of any restrictions on data availability
- For clinical datasets or third party data, please ensure that the statement adheres to our policy

The datasets generated during and/or analysed during the current study are available from the corresponding author on reasonable request.

Field-specific reporting

Please select the one below that is the best fit for your research. If you are not sure, read the appropriate sections before making your selection.

☒ Life sciences ☐ Behavioural & social sciences ☐ Ecological, evolutionary & environmental sciences

For a reference copy of the document with all sections, see nature.com/documents/nr-reporting-summary-flat.pdf

Life sciences study design

All studies must disclose on these points even when the disclosure is negative.

Sample size	Sample size was calculated in bilateral contrast factoring in a 5% alpha risk and 0.1 beta risk (90% statistical power)
Data exclusions	Patients that did not pass this test were excluded from the sample and a nebulizer was recommended for treatment. Other reasons for exclusion were refuse to participate and presence of a language barrier.
Replication	The goodness-of-fit of the multivariate model was evaluated with the Hosmer-Lemeshow test. Odds ratio (OR) values were calculated with 95% confidence intervals (CI 95% CI). All analyses were performed "two tails", and a p-value of less than 0.05 was considered significant.
Randomization	The independent variables considered were: age, sex, Deficient Inhaler Technique (initial visit), Smokers status, Previous training, severity level of disease (COPD / Asthma), type of disease (COPD / Asthma), and Types of inhalers evaluated (initial visit).
Blinding	A blinded researcher carried out the data analysis.

Reporting for specific materials, systems and methods

We require information from authors about some types of materials, experimental systems and methods used in many studies. Here, indicate whether each material, system or method listed is relevant to your study. If you are not sure if a list item applies to your research, read the appropriate section before selecting a response.

Materials & experimental systems

n/a	Involved in the study	
☒	☐	Antibodies
☒	☐	Eukaryotic cell lines
☒	☐	Palaeontology and archaeology
☒	☐	Animals and other organisms
☐	☒	Human research participants
☐	☒	Clinical data
☒	☐	Dual use research of concern

Methods

n/a	Involved in the study	
☒	☐	ChIP-seq
☒	☐	Flow cytometry
☒	☐	MRI-based neuroimaging

Human research participants

Policy information about studies involving human research participants

Population characteristics	The patients included were adults > 18 years of age, suffering from bronchial asthma or COPD, that were being treated with one of the following devices: Pressurized metered-dose inhaler (pMDI) / Soft mist inhaler (SMI), Dry powder inhaler multidose (DPIm), Dry powder inhaler single dose (DPIs) and Pressurized metered-dose inhaler (pMDI) with spacer holding chamber (pMDI+ spacer). The diagnosis of asthma was based on GINA criteria.
Recruitment	Following the recruitment phase, they were scheduled for an initial visit (IV) with a physical therapist who was not involved in the recruitment. Two investigators specializing in pulmonology recruited the patients from the consultation
Ethics oversight	The study protocol was approved by the institutional review board of the hospital, called the "Ethical Committee of Clinical Research of the General University Hospital" on 09/28/2016 (approval number: EST-30/16). All study participants provided written informed consent.

Note that full information on the approval of the study protocol must also be provided in the manuscript.

Clinical data

Policy information about clinical studies

All manuscripts should comply with the ICMJE guidelines for publication of clinical research and a completed CONSORT checklist must be included with all submissions.

Clinical trial registration	Not appliable
Study protocol	Prospective, non-randomized, single-group study, with intervention and before-after evaluation,
Data collection	Between January 11, 2017 and December 21, 2018 with 160 consecutive ambulatory patients of Pneumology a Public General Universitary Hospital
Outcomes	The main variable was the decrease in the percentage of patients with LowAd in the final visit. Secondary variables were: types of non-compliance: erratic unwitting and deliberate, and percentage of patients with poor Inhaler Technique and critical errors.
